# Adverse events of inactivated COVID-19 vaccine in HIV-infected adults

**DOI:** 10.1186/s12981-021-00416-1

**Published:** 2021-12-04

**Authors:** Songjie Wu, Yubin Zhang, Fangzhao Ming, Shi Zou, Mengmeng Wu, Wei Guo, Weiming Tang, Ke Liang

**Affiliations:** 1 Department of Nosocomial Infection Management, Zhongnan Hospital of Wuhan University, Wuhan, Hubei China; 2 Wuchang District Center for Disease Control and Prevention, Wuhan, Hubei China; 3 Department of Infectious Diseases, Zhongnan Hospital of Wuhan University, Wuhan, Hubei China; 4 Department of Pathology, Zhongnan Hospital of Wuhan University, Wuhan, China; 5 Department of Pathology, School of Basic Medical Sciences, Wuhan University, Wuhan, China; 6 Guangdong No. 2 Provincial People’s Hospital, University of North Carolina at Chapel Hill Project-China, Guangzhou, 510095 China; 7 The University of North Carolina at Chapel Hill Project-China, Guangzhou, China; 8 Wuhan Research Center for Infectious Diseases and Cancer, Chinese Academy of Medical Sciences, Wuhan, China; 9 Center of Preventing Mother-To-Child Transmission for Infectious Diseases, Zhongnan Hospital of Wuhan University, Wuhan, China; 10 Department of Nosocomial Infection Management, Department of Infectious DiseasesCenter of Preventing Mother-To-Child Transmission for Infectious Diseases, Wuhan Research Center for Infectious Diseases and CancerChinese Academy of Medical Sciences, Zhongnan Hospital of Wuhan University, Wuhan, 430071 China

**Keywords:** People living with HIV(PLWH), COVID-19, Inactivated vaccine, Adverse event

## Abstract

This study aims to evaluate the safety of inactivated COVID-19 vaccine among adult people living with HIV (PLWH). In total, 259 PLWH who received at least one dose of inactivated COVID-19 vaccine were enrolled, and post-vaccination adverse events (AEs) were evaluated seven days following each vaccination dose. The overall AE frequency was 22.8% after dose one, which was higher than after dose two (10.2%) (*P* < 0.001). No severe side event or vaccine safety concern was observed. Our finding was essential in reducing vaccine hesitancy among PLWH.

People living with HIV (PLWH) tend to have high COVID-19 related morbidity and mortality [1]. UNAIDS suggested that PLWH should be given priority in COVID-19 vaccinations regardless of CD4  +  T lymphocyte count (CD4 count) and HIV viral load (HIV-VL) levels [2]. The Chinese guideline also suggested that PLWH be given the inactivated vaccine or the recombinant subunit vaccine [3]. However, the safety of COVID-19 vaccines among PLWH in China is unknown. This study aims to estimate the adverse events (AEs) rate after COVID-19 vaccination among PLWH.

Between April and July 2021, PLWH from the Wuchang district of Wuhan, China, aged between 18 and 59 years, were enrolled in this study. All participants received inactivated COVID-19 vaccine (Sinopharm, Wuhan Institute of Biological Products Co. Ltd.) on day 0 and day 28 by intramuscular injection. Post-vaccination adverse events were evaluated seven days after each dose of vaccination. These adverse events include injection site pain, swelling, redness, fever, headache, fatigue, drowsiness, and cough.

In total, 91.1% of the PLWH (236/259) have taken both doses, while the remaining 8.9% have only taken the first dose of inactivated vaccine. Of all participants, 99.2% were on antiretroviral therapy (ART), 80.3% were virally suppressed (208/259), and 81.1% had CD4 count  > 350 cells/μl (210/259) at enrollment (Table 1).

**Table 1:** Baseline data for all participants

Characteristic	Patients with HIV/AIDS (n = 259)	Dose 1(N = 259)				Dose 2 (N = 236)			
		With adverse events	Without adverse events	χ^2^	*P*	With adverse events	Without adverse events	χ^2^	*P*
Age group									
≤ 35	116	30	86			11	89		
> 35	143	29	114	1.14	0.29	13	123	0.13	0.72
Gender									
Male	240	55	185			21	197		
Female	19	4	15	0.04	0.85	3	15	0.90	0.34
Marital status									
Singe	169	42	127			17	136		
Married	36	6	30			3	31		
Other	54	11	43	1.36	0.51	4	45	0.43	0.81
Occupation									
Unemployment	37	7	30			2	33		
Employment	222	52	170	0.37	0.55	22	179	0.89	0.35
Education level									
High school or lower	76	17	59			6	62		
Higher than high school	183	42	141	0.01	0.92	18	150	0.19	0.66
Comorbidities									
Yes	38	6	32			5	30		
No	221	53	168	1.24	0.27	19	182	0.76	0.38
NNRTIs (NVP/EFV)									
Yes	216	47	169			17	180		
No	43	12	31	0.77	0.38	7	32	–	0.09
INSTIs (EVG/DTG)									
Yes	26	5	21			1	22		
No	233	54	179	0.21	0.65	23	190	–	0.48
PIs (LPV/r)									
Yes	14	5	9			5	8		
No	245	54	191	0.74	0.39	19	204	9.00	0.003
CD4									
≤ 350	49	8	41			5	38		
> 350	210	51	159	1.43	0.23	19	174	0.12	0.73
HIV viral load									
Undetectable	208	49	159			19	172		
Detectable	51	10	41	0.36	0.55	5	40	–	0.79

*NNRTIs* nonnucleoside reverse transcriptase inhibitors; *INSTIs* integrase inhibitors; *PIs* protein inhibitors; *NVP* nevirapine; *EFV* efavirenz; *EVG* elvitegravir; *DTG* dolutegravir; *LPV/r* lopinavir/ritonavir; *–* no statistics are computed because Fisher exact method was used

The overall AE rate was 22.8% after dose one (D1) of the vaccination, which was higher than that after dose two (10.2%) (P  < 0.001). Local injection-site reactions were reported in 17.0% of the participants after D1 and 7.6% after D2. The most common systemic reactions included fatigue (3.5% after D1, and 0.8% after D2, drowsiness (2.3% after D1, and 1.7% after D2), fever (1.9% after D1, and 0.0% after D2) (Fig. 1).

**Fig. 1:**
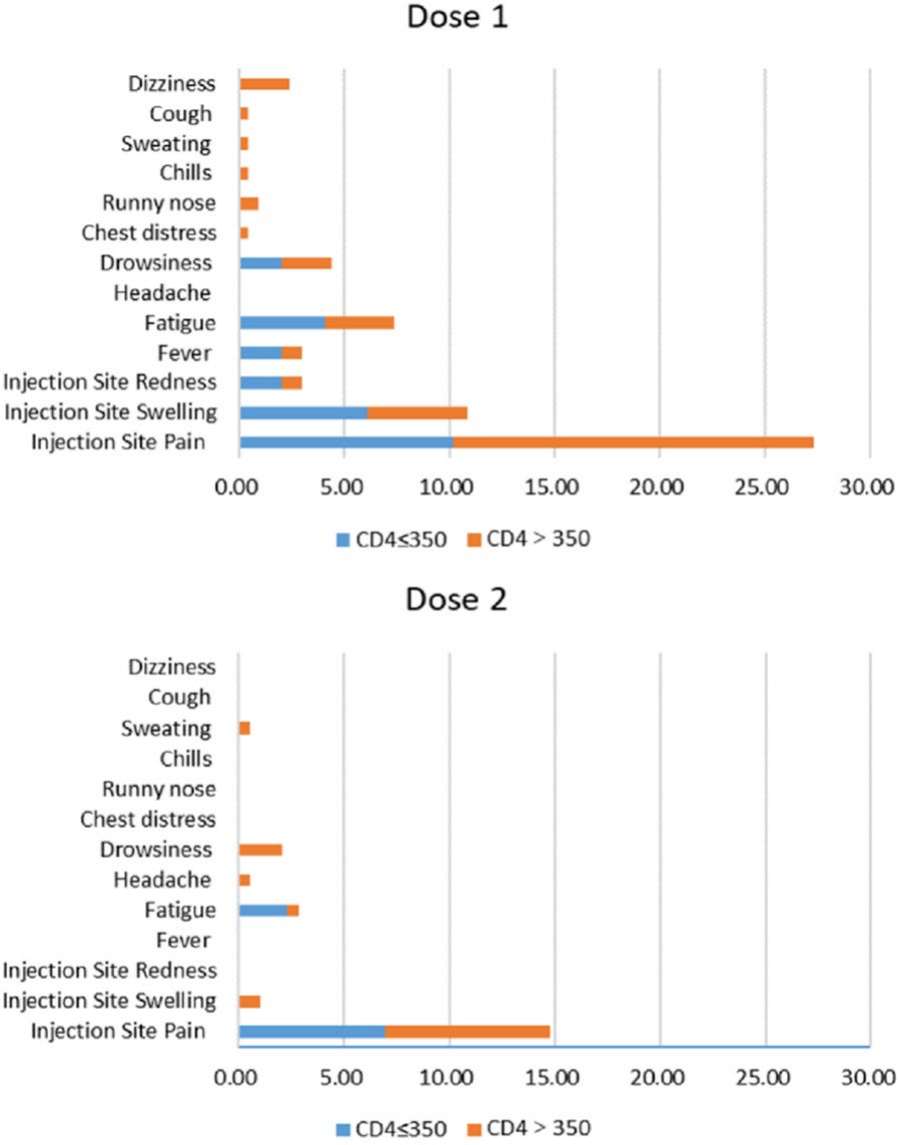
Adverse events after each vaccine dose stratified by CD4 count

The majority of AEs were non-severe. The most common severe symptom after D1 included fatigue (3.1%), drowsiness (2.3%), and dizziness (1.9%). The most common severe symptom after D2 was drowsiness (1.7%). No other severe adverse events were observed. Compared with participants with other ART regimens (7.6%), participants receiving protein inhibitor (PI) based antiretroviral regimen (all PI is lopinavir/ritonavir) reported more AEs (38.5%) after D2 (*P*  < 0.05). No significant differences in any AE rates were observed in other subgroups of PLWH (*P*  > 0.05). After adjusted for age, sex, comorbidities, CD4 count, and HIV viral load with multivariable logistic regression model, receiving LPV/r based regimen were still associated with increased AE risk in D2 (OR  = 11.92, 95% CI 2.63–54.00; *P*  = 0.001) (Table 2). We also found no difference in AE rates after each dose between participants with CD4  > 350/μL and  ≤ 350/μL (*P * > 0.05).

**Table 2:** Risk factors associated with AEs of inactivated COVID-19 vaccine in HIV-infected adults after dose 1 and dose 2: multivariable logistic regression analysis

Item	Dose 1		Dose 2	
	Adjusted OR^a^ (95% CI)	*P*	Adjusted OR^a^ (95% CI)	*P*
Age				
≤ 35	Ref.		Ref.	
> 35	0.68 (0.37, 1.26)	0.22	0.58 (0.22, 1.49)	0.26
Gender				
Male	Ref.		Ref.	
Female	1.03 (0.32, 3.34)	0.96	2.54 (0.62, 10.41)	0.20
Comorbidities				
No	Ref.		Ref.	
Yes	0.66 (0.26, 1.70)	0.39	1.85 (0.60, 5.71)	0.28
PIs (LPV/r)				
No	Ref.		Ref.	
Yes	2.97 (0.86, 10.30)	0.09	11.92 (2.63, 54.00)	0.001
CD4				
≤ 350	Ref.		Ref.	
> 350	1.71(0.72,4.09)	0.23	1.26 (0.39, 4.13)	0.70
HIV viral load				
Undetectable	Ref.		Ref.	
Detectable	0.74 (0.32, 1.69)	0.47	0.58 (0.15, 2.24)	0.43

^a^Each association was mutually adjusted for the other characteristics in the table

Concerns around AEs significantly impact ongoing vaccine hesitancy among PLWH. A previous national survey found that about 37.1% of PLWH are concerned that COVID-19 vaccination may have severe side effects [4]. Our study extended the existing literature by reporting AEs after COVID-19 vaccination among PLWH [5–7]. In our study, the AE rates were 22.8% after dose one (D1) of the inactivated COVID-19 vaccination and 10.2% after dose two, which was not higher than the AE rates of the original inactivated COVID-19 vaccine trials in general population [8, 9]. The AE rates of inactivated COVID-19 vaccine in our study was lower than that of mRNA COVID-19 vaccine [5, 6, 10] and adenovirus vector COVID-19 vaccine [7]. We conclude the adverse events after the two-dose of inactivated COVID-19 vaccination among PLWH are minimal and mild. In addition, we also found that participants who were receiving LPV/r based regimen were more likely to experience AE after D2.

Our results have direct and immediate clinical implications. The data in this analysis are reassuring, finding no severe adverse event or vaccine safety concern among PLWH. There is an urgent need to disseminate this information to the vulnerable group of PLWH to minimize vaccine hesitancy and eliminate its refusal.

## Data Availability

All data generated or analyzed during this study are included in this article. The datasets generated and analyzed during the current study are available from the corresponding author on reasonable request.
