# Correction: Adverse events of inactivated COVID-19 vaccine in HIV-infected adults

**DOI:** 10.1186/s12981-024-00654-z

**Published:** 2024-09-28

**Authors:** Songjie Wu, Yubin Zhang, Fangzhao Ming, Shi Zou, Mengmeng Wu, Wei Guo, Weiming Tang, Ke Liang

**Affiliations:** 1 Department of Nosocomial Infection Management, Zhongnan Hospital of Wuhan University, Wuhan, Hubei China; 2 Wuchang District Center for Disease Control and Prevention, Wuhan, Hubei China; 3 Department of Infectious Diseases, Zhongnan Hospital of Wuhan University, Wuhan, Hubei China; 4 Department of Pathology, Zhongnan Hospital of Wuhan University, Wuhan, China; 5 Department of Pathology, School of Basic Medical Sciences, Wuhan University, Wuhan, China; 6 Guangdong Second Provincial General Hospital, Guangzhou, China; 7 The University of North Carolina at Chapel Hill Project-China, Guangzhou, China; 8 Wuhan Research Center for Infectious Diseases and Cancer, Chinese Academy of Medical Sciences, Wuhan, China; 9 Center of Preventing Mother‑To‑Child Transmission for Infectious Diseases, Zhongnan Hospital of Wuhan University, Wuhan, China; 10 Department of Nosocomial Infection Management, Department of Infectious Diseases Center of Preventing Mother-To-Child Transmission for Infectious Diseases, Wuhan Research Center for Infectious Diseases and Cancer Chinese Academy of Medical Sciences, Zhongnan Hospital of Wuhan University, Wuhan, 430071 China

**Correction to: AIDS Research and Therapy (2021) 18:92**

10.1186/s12981-021-00416-1

In this article [1], the affiliation details for the Author Weiming Tang were incorrectly given as “Guangdong No. 2 Provincial People’s Hospital, University of North Carolina at Chapel Hill Project-China, Guangzhou 510095, China” but should have been “Guangdong Second Provincial General Hospital, Guangzhou, China”.
